# MoS_2_ Heterojunctions by Thickness Modulation

**DOI:** 10.1038/srep10990

**Published:** 2015-06-30

**Authors:** Mahmut Tosun, Deyi Fu, Sujay B. Desai, Changhyun Ko, Jeong Seuk Kang, Der-Hsien Lien, Mohammad Najmzadeh, Sefaattin Tongay, Junqiao Wu, Ali Javey

**Affiliations:** 1 Electrical Engineering and Computer Sciences, University of California, Berkeley, CA, 94720; 2 Materials Sciences Division, Lawrence Berkeley National Laboratory, Berkeley, CA 94720; 3 Berkeley Sensor and Actuator Center, University of California, Berkeley, CA, 94720; 4 Department of Materials Science and Engineering, University of California, Berkeley, CA, 94720.

## Abstract

In this work, we report lateral heterojunction formation in as-exfoliated MoS_2_ flakes by thickness modulation. Kelvin probe force microscopy is used to map the surface potential at the monolayer-multilayer heterojunction, and consequently the conduction band offset is extracted. Scanning photocurrent microscopy is performed to investigate the spatial photocurrent response along the length of the device including the source and the drain contacts as well as the monolayer-multilayer junction. The peak photocurrent is measured at the monolayer-multilayer interface, which is attributed to the formation of a type-I heterojunction. The work presents experimental and theoretical understanding of the band alignment and photoresponse of thickness modulated MoS_2_ junctions with important implications for exploring novel optoelectronic devices.

Semiconducting transition metal dichalcogenides (TMDCs) with a layered crystal structure exhibit unique electrical^1^,^2^ and optical properties^3^,^4^,^5^. TMDCs provide opportunities in exploring new device concepts given their atomic level flatness, and ability to form van der Waals (vdW) heterostructures with strong interlayer coupling^6^,^7^,^8^. For instance, vdW heterobilayers of MoS_2_/WSe_2_ have been recently reported to exhibit spatially *direct* light absorption but spatially *indirect* light emission, representing a highly intriguing material property^9^,^10^. Here, we explore the optoelectronic properties of lateral “hetero”-junctions formed on a single crystal of MoS_2_ of varying thickness (i.e., number of layers). As a result of the quantum confinement effect^11^, when the thickness of a MoS_2_ crystal is scaled down to a monolayer the optical band gap increases from 1.29 eV (indirect) to 1.85 eV (direct)^12^,^13^. The change in the band structure and the electron affinity of MoS_2_ with layer number opens up the path to the formation of atomically sharp heterostructures, not by changing composition but rather by changing layer thickness^14^. We experimentally examine the surface potential of this thickness modulated heterojunction by using Kelvin probe force microscopy (KPFM). We further use scanning photocurrent microscopy (SPCM) to probe the photoresponse of the junction. A large photocurrent response is observed at the monolayer/multilayer junction interface which confirms the presence of a strong built-in electric field at the interface. Device modeling is used in parallel to experiments to understand the underlying mechanism of the observed photocurrents and the band-alignments at the junction interface, suggesting the formation of a type-I heterojunction.

SPCM has been previously used to study the photoresponse of metal contacted MoS_2_ transistors, where the channel thickness for MoS_2_ was uniform throughout the device^15^,^16^. The results have shown that the photoresponse is primarily driven by the metal/MoS_2_ Schottky contacts and photothermoelectric effect^16^. In distinct contrast to previous studies, we observe that the peak photoresponse is spatially located at the MoS_2_ monolayer/multilayer junction for our lateral heterojunctions and not at the metal contacts.

## Results

### Band offset extraction at the monolayer-multilayer MoS_2_ junction

**Figure 1:**
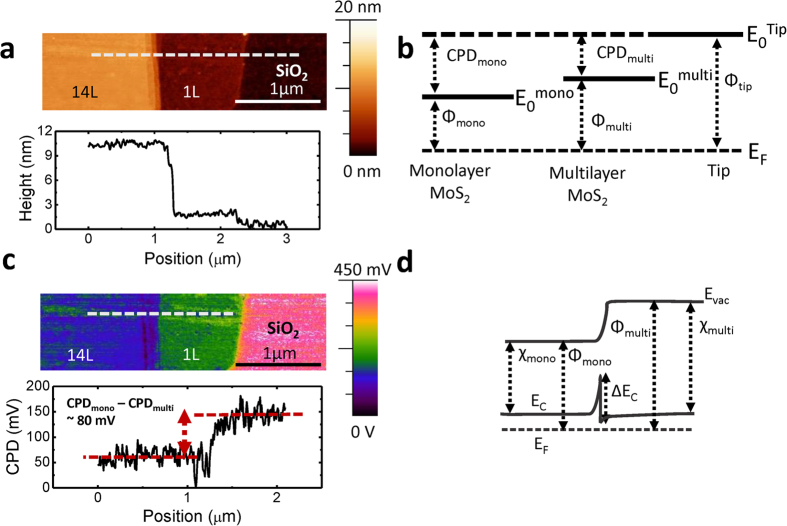
**a**. Atomic Force Microscope (AFM) image of a monolayer-multilayer MoS_2_ flake. **b.** Representative energy band diagrams of isolated monolayer and multilayer MoS_2_ with respect to the AFM tip, depicting CPD and work function values. **c.** Kelvin Force Probe Microscope (KPFM) image of a representative 1L-14L MoS_2_ flake. **d.** Representative band diagram of a monolayer-multilayer device at equilibrium.

KPFM is performed to spatially map the surface potential, and shed light on the band offsets at the monolayer-multilayer interface. The sample surface topography and contact potential difference (CPD) between the tip and sample are measured simultaneously^17^,^18^. Figure 1a demonstrates a monolayer-multilayer junction flake with 10 nm of multilayer (~14 layers; 14 L) thickness. In this particular flake, monolayer to multilayer transition occurs across ~100 nm of the lateral distance in a terraced manner. KPFM is performed using a Bruker MultiMode atomic force microscope under ambient conditions. A Si cantilever tip coated with Pt-Ir (SCM-PIT, Bruker Co.) is used in the tapping mode. Electrical contacts to the MoS_2_ flake were grounded during the measurements. An AC voltage of 2 V is applied to the tip while the tip height is kept constant at 5 nm. The measured DC voltage of the tip, corresponding to CPD, determines the work function difference between the AFM tip (Pt-Ir) and each region of the MoS_2_ flake^19^,^20^, i.e., 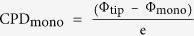 for the monolayer side and 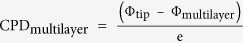 for the multilayer side. Φ_mono_, Φ_multilayer_, and Φ_tip_ are the work functions of monolayer MoS_2_, multilayer MoS_2_ and the surface of AFM tip, respectively (Fig. 1b). The measured surface potential difference, 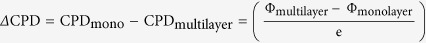, corresponds to the band bending in the vacuum level *E*_vac_ at thermal equilibrium, and is also equal to the workfunction difference between the monolayer and the multilayer (Fig. 1b). KPFM map of a representative 1 L–14 L flake is shown in Fig. 1c. From KPFM measurements, the workfunction difference is found to be ~80 meV (Fig. 1c)^21^,^22^. Next, we focus on obtaining the energy band diagram for the heterojunction by first extracting the conduction band offset at the interface. The conduction band offset 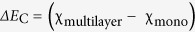 at the heterojunction corresponds to the electron affinity difference between the monolayer (χ_mono_) and multilayer (χ_multilayer_). The workfunction difference between monolayer and multilayer is related to effective density of states (*N*_C_), 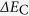 and doping levels (*N*_D_) as shown in Eq. 1. Here, *k* is the Boltzmann constant and *T* is the temperature.


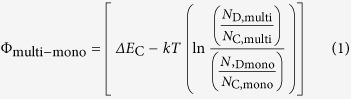


Boltzmann approximation is considered while deriving Eq. 1. Next we assume the doping level per unit volume is identical in both monolayer and multilayer flakes. Thus Eq. 1 becomes,


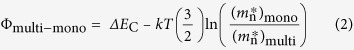


Φ_multi−mono_ is measured from KPFM, effective mass values for electrons are taken to be m^*^_n,mono_ = 0.407 m_0_ and m^*^_n,multi_ = 0.574 m_0_. From these parameters, ∆*E*_C_ of ~70 meV at the 1 L–14 L interface is extracted. This band offset corresponds to a type-I heterojunction as depicted in the qualitative band diagram of Fig. 1d. Note that the relative values of the dielectric constant of monolayer and multilayer MoS_2_ determine the electric fields and hence the band bending on both sides of the heterojunction. The dielectric constants are assumed to be the same (~4) in this work^24^.

### Photoresponse at the monolayer-multilayer MoS_2_ junction

**Figure 2:**
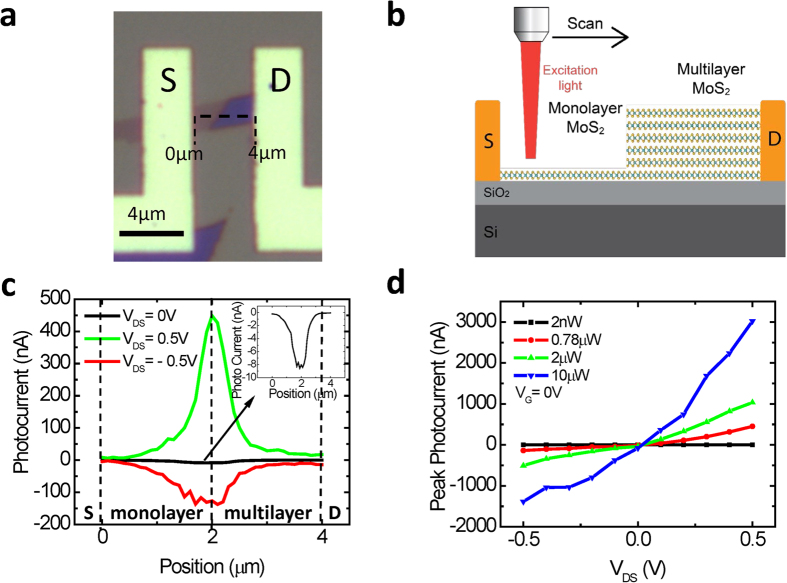
**a**. Optical microscope image of the monolayer-multilayer device with Ni/Au (30 nm/30 nm) contacts. **b.** Schematic representation of the SPCM measurement. **c.** Photoresponse of the monolayer-multilayer MoS_2_ flake versus position along the dashed line at *V*_G_ = 0 V, with illumination power of 0.78 μW and with *V*_DS_ = −0.5 V, *V*_DS_ = 0.5 V and *V*_DS_ = 0 V. **d.** Peak photocurrent vs. V_DS_ at V_GS_ = 0 V with different illumination powers.

Scanning photocurrent microscopy (SPCM), a spatially resolved photodetection technique that combines electrical measurement and local illumination with a focused laser beam is used to probe the local photoresponse of the monolayer-multilayer MoS_2_ devices^25^,^26^,^27^,^28^,^29^,^30^. Optical image of a representative device is shown in Fig. 2a. Here, the device consists of a 1 L–5 L MoS_2_ junction. The channel lengths for the monolayer and the multilayer regions are ~2 μm each. The contact to the monolayer is defined as the source electrode and is electrically grounded. The contact to the multilayer MoS_2_ serves as the drain electrode to which an external voltage, *V*_DS_ is applied during the measurements. The heavily doped Si substrate serves as the global back-gate to which voltage, *V*_G_ can be applied to modulate the electric potential in MoS_2_. The device is locally illuminated by a focused laser beam (wavelength: 488 nm, diameter: ~1 μm) as depicted in Fig. 2b. The spatial resolution of the scanning stage is 0.1 μm. The light current, *I*_light_ is recorded as the laser spot is scanned across the length of the device. The photocurrent, *I*_photocurrent_, is then obtained as a function of illumination spot by subtracting the dark current of the device, *I*_dark_, from *I*_light_. In contrast to previous reports studying the photoresponse in MoS_2_ single layer or multilayer devices (Fig. S2)^3^,^15^,^16^,^31^, the peak photocurrent in our device is observed at the monolayer-multilayer interface rather than the metal/semiconductor junction. Figure 2c shows the spatial response of the photocurrent along the dashed line of Fig. 2a at *V*_G_ = 0 V and laser intensity of 0.78 μW. Source-drain voltage is varied from −0.5 V to 0.5 V. Even without source drain bias (*V*_DS_ = 0 V), a finite short circuit current (~8 nA) is measured as seen in the inset of Fig. 2c. This implies that the expected band bending and the resulting built-in electric field that is induced by the difference in the electron affinities of the monolayer and multilayer MoS_2_ regions is capable of separating the electron-hole pairs generated at the monolayer-multilayer interface. The dependence of the photocurrent on *V*_DS_ and the excitation power is investigated to further characterize the monolayer-multilayer junction devices. The peak photocurrent, corresponding to the local illumination of the monolayer-multilayer interface, is measured at different illumination intensities of 2 nW, 0.78 μW, 2 μW and 10 μW and *V*_DS_ of −0.5 V to 0.5 V at *V*_GS_ = 0 V (Fig. 2d). The photocurrent increases as the applied *V*_DS_ bias is increased due to the contribution of the enhanced drift current and decreased transit time of the electrons^3^. The increase in the photocurrent with the illumination can be attributed to generation of higher number of electron-hole pairs. It is important to note that at all different laser powers and *V*_DS_ values the peak photocurrent response is observed at the monolayer-multilayer heterojunction.

### Photoresponsivity at the monolayer-multilayer MoS_2_ junction

**Figure 3:**
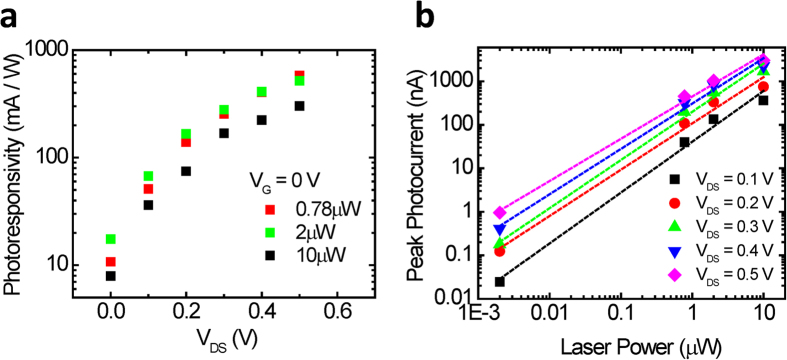
**a**. Photoresponsivity vs. the applied V_DS_ at V_G_ = 0 V and with different laser power. **b.** Peak photocurrent vs. laser power at *V*_G_ = 0 V and at different *V*_DS_ values.

To further characterize the photocurrent generation at the heterojunction, photoresponsivity is experimentally investigated as a function of *V*_DS_ and laser power. Photoresponsivity determines the gain of a photodetector system in terms of the ratio of photocurrent generated (*I*_photocurrent_) and the incident laser power (*P*_incident_), i.e., (*I*_photocurrent_) / *P*_incident_. Figure 3a shows the photoresponsivity with varying laser powers at *V*_G_ = 0 V. It is found to increase as the *V*_DS_ increases from 0 V to 0.5 V. The maximum photoresponsivity is found to be 580 mA / W at *V*_DS_ = 0.5 V and with the power of 0.78 μW. Given the power of the laser (0.78 μW) and the *V*_DS_ value of 0.5 V and at *V*_G_ = 0 V, the maximum photoresponsivity of the monolayer – multilayer heterojunction is found to be greater than the highest reported MoS_2_ photodetector in the literature^3^,^15^,^31^,^32^. Moreover, the dependence of the photocurrent on power is investigated. Figure 3b shows a linear relationship between the peak photocurrent and the laser power. This is consistent with the response of standard photodiodes where the photocurrent is proportional to the carrier generation rate and hence the light intensity^33^.

### Dependence of the peak photocurrent on the gate bias

**Figure 4:**
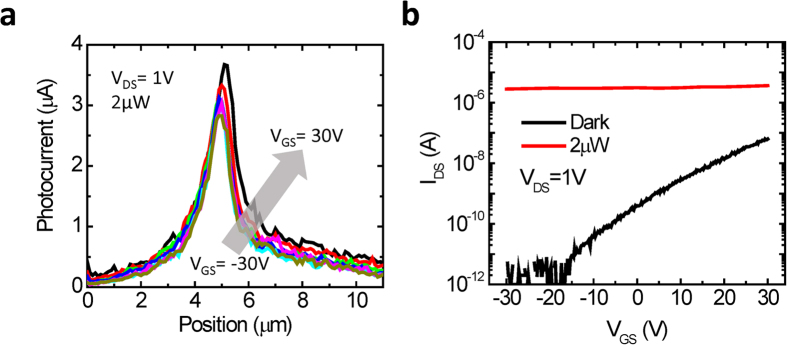
**a**. Photocurrent vs. position at V_DS_ = 1 V with illumination power of 2 μW and V_GS_ varied from −30 V to 30 V at 10 V increments. **b.** *I*_DS_ vs. *V*_GS_ at *V*_DS_ = 1 V in dark and with 2 μW of illumination power.

Next, we explore the effect of gate voltage on the peak photocurrent. Figure 4a shows the measured photocurrent as a function of displacement along the length of the device for *V*_GS_ ranging from −30 V to 30 V. The drain voltage is maintained constant at 1 V with an illumination power of 2 μW. The effect of gate voltage on the photocurrent is minimal. This is further illustrated in Fig. 4b where the peak photocurrent is plotted as a function of gate voltage. For comparison the dark current as a function of gate voltage for the same device is also shown. While the dark current shows strong gate dependency, consistent with n-type characteristics of MoS_2_, the light current exhibits nearly no gate dependence. This is distinct from the previous studies of photocurrent for a uniform thickness MoS_2_ flake, where the gate voltage was shown to modulate the Schottky barrier heights and thus the photoresponse^34^. In contrast, the peak photocurrent in our devices arise from the monolayer-multilayer junction where the global back-gate has minimal effect on its potential profile.

## Discussion

**Figure 5:**
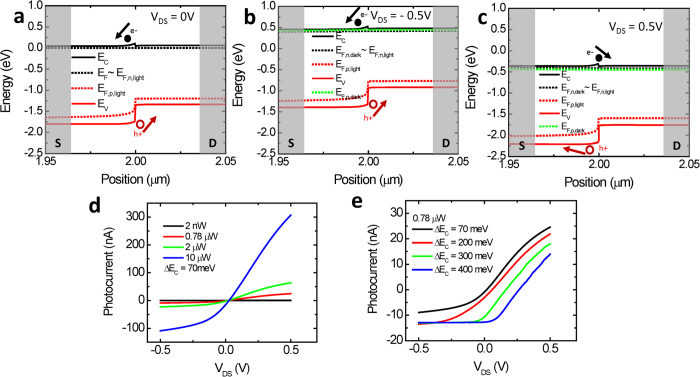
Simulated band diagrams at **a**, V_DS_ = 0 V, **b,** V_DS_ = −0.5 V and **c**, VDS = 0.5 V in dark and with light shined at the monolayer – multilayer MoS_2_ junction. **d.** Simulated photocurrent vs. *V*_DS_ at different laser powers. **e.** Simulated photocurrent vs. *V*_DS_ with different ∆*E*_C_ values.

Device modeling was performed using TCAD Sentaurus to further understand the junction properties for a monolayer-multilayer device. For the Sentaurus simulations a ∆*E*_C_ ~ 70 meV, calculated from the KPFM data is used. The doping level for both monolayer and multilayer regions are assumed to be *N*_D,mono_ = *N*_D,multi_ = 10^18^ cm^−3,^^35^. The effect of the back gate is modeled as a change in the device doping concentration. The effective mass values are taken to be m^*^_e_ ~ 0.407 m_0_ for the monolayer and m^*^_e_ ~ 0.574 m_0_ for the multilayer as described earlier^23^. The dielectric constants are assumed to be the same ε_mono_ = ε_multilayer_ = 4 ε_0_^23^. For the simulated device, the exact dimensions of the measured device presented in Fig. 2 are used. Electron affinities are assumed to be χ_monolayer_ = 4 eV^36^ and χ_multilayer_ = 4.07 eV, such that ∆*E*_C_ = 70 meV as obtained earlier using the experimental KPFM data. Measured values for bandgap of monolayer (1.85 eV) and 5 layers (1.4 eV) are used^12^. A light window of 1 μm is used that corresponds to the spot size of the laser and is shined in the center of the junction. A laser wavelength of 488 nm and an absorption coefficient of 10^6^ cm^−1^ for the monolayer and 10^5^ cm^−1^ for the multilayer side is assumed for the simulations^15^,^37^. Thus with the assumptions stated above and using the KPFM information, simulations revealed a type – I heterojunction band alignment in the monolayer – multilayer MoS_2_ heterojunction flake as seen in Fig. 5a–c.

Figure 5a shows the simulated band diagram at V_DS_ = 0 V for the dark condition and when light is illuminated at the monolayer-multilayer interface. Under illumination, Fermi levels split (E_F,n_ and E_F,p_) as a result of the generation of the electron hole pairs. At a simulated laser power of 0.78 μW, low level injection conditions prevail and no change in the quasi Fermi level for electrons is observed as seen in Fig. 5a. At zero *V*_DS_ bias, due to the built-in electric field at the heterojunction, electrons that are generated at the monolayer side of the monolayer-multilayer junction are swept to the monolayer side (source). However electrons generated at the multilayer side are subjected to a barrier height of 70 meV. Holes do not encounter any barrier and freely move to the multilayer side (drain). This is consistent with the measured SPCM data, where a negative photocurrent of 8 nA is recorded at zero *V*_DS_ signifying that the electrons are collected at the source (monolayer) and holes at the drain (multilayer) side.

Previous studies have shown small Schottky barrier heights for electrons both in monolayer and multilayer MoS_2_, on the order of 200 meV or less^38^,^39^,^40^,^41^. The barrier height for electrons at the monolayer-multilayer interface is ∆*E*_C_ = 70 meV as calculated. In this system, the Schottky barrier height at the contacts and the barrier height at the junction are on the same order of magnitude. Therefore a part of the applied VDS gets dropped at the contacts, and the remaining voltage is dropped at the monolayer-multilayer MoS_2_ interface. When a negative *V*_DS_ (Fig. 5b) is applied to the multilayer side (drain), there is a higher built-in electric field and a wider depletion region at the junction. A wider depletion region at the monolayer side allows the separation of a larger number of photogenerated electron-hole pairs thus resulting in a larger negative photocurrent compared to the case of zero *V*_DS_ as the electrons get swept to the monolayer side (source) and holes swept to the multilayer side (drain) freely. However electrons generated at the multilayer side still face a barrier height of ~70 meV, just like in the case of zero bias. By applying a positive V_DS_ (Fig. 5c) the barrier height for electron transport from the monolayer to the multilayer is nearly diminished. Electron-hole pairs generated at the monolayer side contribute to the photocurrent since holes move to the monolayer side (source) freely and electrons can go over the decreased barrier height and move to the multilayer side (drain). Whereas, electron-hole pairs generated at the multilayer side don’t contribute to the photocurrent since holes see a barrier of Δ*E*_V_ (~0.38 eV). This current flow mechanism is consistent with measuring positive photocurrent at *V*_DS_ > 0 and measuring negative photocurrent for *V*_DS_ < 0 as seen in Fig. 2d.

Photocurrent vs. applied V_DS_ is also simulated in TCAD Sentaurus with different illumination intensities. The same parameters and assumptions that are used to generate the band diagrams mentioned above are used to simulate the *V*_DS_ and the light intensity dependence of the photocurrent. In the SPCM measurements, as seen in Fig. 2c, at *V*_DS_ = 0 V a negative photocurrent is observed. As the applied bias is increased to *V*_DS_ = 0.1 V, photocurrent becomes positive (Fig. 2d). This implies that the experimental crossover from the negative photocurrent to positive photocurrent is in between *V*_DS_ = 0 V and *V*_DS_ = 0.1 V. The simulated photocurrents are shown in Fig. 5d–e. The simulation is in qualitative agreement with the experimental data, with the transition from the negative to positive photocurrent occurring at positive *V*_DS_ values. The negative to positive photocurrent crossover voltage is sensitive to the parameter values assumed for the simulations. Figure 5e illustrates the large dependence of the simulated crossover voltage on the ∆*E*_C_ value. Quantitative differences between the simulated and experimental data can also arise from the presence of a terraced junction as described in Fig. 1a, compared to the ideal step heterojunction simulated in Sentaurus. The Sentaurus simulations however qualitatively explain the experimental data and all the trends, but a quantitative analysis warrants simulations or first principle calculations using exact values of absorption coefficient, electron affinities, effective masses, doping, carrier lifetimes, diffusion lengths, etc.

In conclusion, the type-I heterojunctions enabled by lateral thickness modulation of MoS_2_ are demonstrated. The junction properties are characterized by KPFM and SPCM. A workfunction difference of 80 meV is measured by KPFM. Furthermore, a conduction band offset of 70 meV is extracted from the difference in the electron affinities and work functions of the monolayer and multilayer regions of the MoS_2_. Photocurrent generation at the monolayer-multilayer heterojunction is observed with SPCM. The peak photocurrent generation at the monolayer-multilayer junction is attributed to the electric field in the depletion region at the heterojunction formed by the difference in the work functions and the electron affinities of the monolayer and the multilayer flake. A short circuit current of 8 nA is measured due to the built-in electric field being able to separate and collect the generated electron hole pairs at the monolayer-multilayer junction. The photoresponsivity of the monolayer-multilayer MoS_2_ junction is studied with respect to the incident light power and the source-drain bias. The demonstration of the type-I heterojunction on the same MoS_2_ flake will inspire further investigation regarding the electronic transport properties of the atomically sharp type-I band alignment in the TMDC flakes.

## Methods

The fabrication process for thickness modulated MoS_2_ heterojunction devices is as follows. MoS_2_ crystals (SPI Supplies) are transferred onto Si/SiO_2_ (260 nm thick) substrates using the micromechanical exfoliation technique. The flakes of interest consisting of mono-multilayer junctions are identified using an optical microscope. These flakes are formed naturally during the exfoliation process. In order to verify the thicknesses of the mono-multilayer regions, atomic force microscopy (AFM) is performed (Fig. 1a). Monolayer thickness of 0.7 nm is confirmed^1^ and a multilayer thicknesses ranging from 6–15 nm is measured for the different samples explored in this study. Photoluminescence (PL) mapping of the flakes was conducted to further depict the mono- and multi-layer regions using a 532 nm pump laser with 8–80 μW power and a spot size of ∼0.5 μm (Horiba Scientific LabRAM HR 800). PL map of a representative flake is shown in Fig. S1c, where the luminescence signal ratio is approximately one order of magnitude between the two regions of the MoS_2_, further depicting the formation of a thickness modulated heterojunction. Metal source/drain (S/D) contacts are subsequently formed with one contact on the monolayer region and the other on the multilayer region of the MoS_2_ flake. Electron-beam lithography was used to pattern the metal contacts, followed by evaporation of Ni/Au (30/30 nm), and lift-off of the resist in acetone.

## Additional Information

**How to cite this article**: Tosun, M. *et al.* MoS_2_ Heterojunctions by Thickness Modulation. *Sci. Rep.* **5**, 10990; doi: 10.1038/srep10990 (2015).

## Supplementary Material

Supplementary Information
