# Supplementary material for: MoS2 Heterojunctions by Thickness Modulation
Source: Sci Rep. 2015 Jun 30;5:10990. doi: 10.1038/srep10990 (PMC4485222; doi:10.1038/srep10990)
Supplement: Supplementary Information [file srep10990-s1.pdf]

MoS_2 Heterojunctions by Thickness Modulation

Mahmut Tosun, [1,2,3*] Deyi Fu,[2,4*] Sujay B. Desai,[1,2,3,*] Changhyun Ko,[4] Jeong Seuk Kang,[1,2,3] Der-Hsien Lien,[1,2,3] Mohammad Najmzadeh,[1,3] Sefaattin Tongay,[4] Junqiao Wu,[2,4] Ali Javey[1,2,3,†]

[1]Electrical Engineering and Computer Sciences, University of California, Berkeley, CA, 94720.

[2]Materials Sciences Division, Lawrence Berkeley National Laboratory, Berkeley, CA 94720.

[3]Berkeley Sensor and Actuator Center, University of California, Berkeley, CA, 94720.

[4]Department of Materials Science and Engineering, University of California, Berkeley, CA, 94720.

† Corresponding Author: ajavey@eecs.berkeley.edu

* Equally contributing authors

Characterization of exfoliated monolayer – multilayer MoS_2 flakes

Figure S1a shows the optical image of an as exfoliated mono – multilayer MoS_2 flake. After optically detecting the flake, AFM is done to confirm the thicknesses of the different regions of the flake. Figure S1b shows the AFM image of an exfoliated flake where 0.7nm of monolayer thickness is measured. Multilayer flakes in contact with the monolayer flake are detected starting from 6 nm and increasing in thickness. Figure S1c shows the photoluminescence (PL) mapping of the monolayer – multilayer flake. Due to the indirect band gap of the multilayer flake and the direct band gap of the monolayer flake, a one order of magnitude higher signal is detected from the monolayer clearly identifying the monolayer part of the mono – multilayer MoS_2 flake.

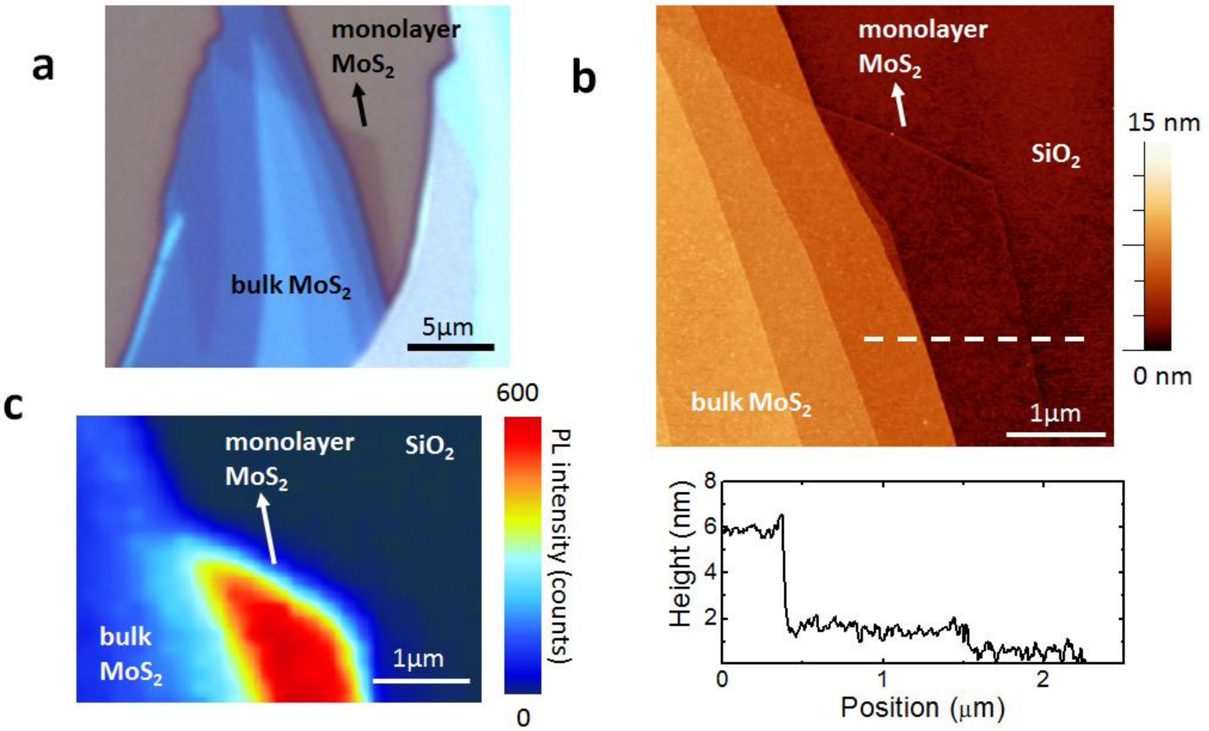

Figure S1a. Optical image of an as exfoliated mono-multilayer MoS_2 flake. **b.** Atomic Force Microscope (AFM) image of the corresponding mono-multilayer MoS_2 flake. **c.** Photoluminescence (PL) mapping of the mono-multilayer MoS_2 flake.

Control SPCM measurements done on a multilayer MoS_2 flake

In order to verify the spatial response of the peak photocurrent from the center of the mono-multilayer heterojunction flake, a control SPCM measurement is done on a multilayer MoS_2 flake. As seen in Fig. S2, the peak photoresponse from the uniform thickness MoS_2 flake is obtained from the close proximity to the contacts of the device that is consistent with the literature. [1, 2]

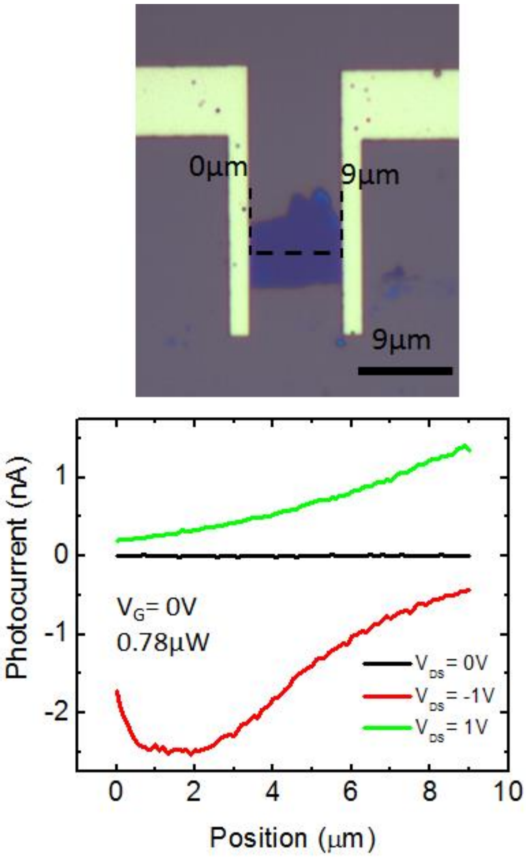

Figure S2. SPCM measurement of a multilayer MoS_2 flake with uniform thickness.

References

1. Wu, C.-C. *et al*. Elucidating the Photoresponse of Ultrathin MoS_2 Field-Effect Transistors by Scanning Photocurrent Microscopy. *J. Phys. Chem. Lett.* **4**, 2508-2513 (2013).

2. Lopez-Sanchez, O., Lembke, D., Kayci, M., Radenovic, A., Kis, A. Ultrasensitive photodetectors based on monolayer MoS_2. *Nat Nano*. **8**, 497-501 (2013).
